# Continuity of care and mortality in patients with type 2 diabetes: a cohort study

**DOI:** 10.3399/BJGPO.2024.0144

**Published:** 2025-01-29

**Authors:** Eero H Mellanen, Timo Kauppila, Hannu Kautiainen, Mika T Lehto, Ossi Rahkonen, Kaisu H Pitkälä, Merja K Laine

**Affiliations:** 1 Department of General Practice and Primary Health Care, University of Helsinki and Helsinki University Hospital, Helsinki, Finland; 2 Folkhälsan Research Centre, Helsinki, Finland; 3 Primary Health Care Unit, Kuopio University Hospital, Kuopio, Finland; 4 City of Vantaa, Vantaa, Finland; 5 Department of Public Health, University of Helsinki, Helsinki, Finland

**Keywords:** continuity of patient care, family medicine, diabetes mellitus, primary healthcare

## Abstract

**Background:**

How GP continuity of care (GP-CoC) affects mortality in patients with type 2 diabetes (T2D) is unclear.

**Aim:**

To examine the effect of having no continuity of care (CoC) and GP-CoC on mortality in primary health care (PHC) patients with T2D.

**Design & setting:**

A cohort study in patients aged ≥60 years with T2D, which was conducted within the public PHC of the city of Vantaa, Finland.

**Method:**

The inclusion period was between 2002 and 2011 and follow-up period between 2011 and 2018. Six groups were formed (no appointments, one appointment and Modified, Modified Continuity Index [MMCI] quartiles). Mortality was measured with standardised mortality ratio (SMR) and adjusted hazard ratio (aHR). GP-CoC was measured with MMCI. Comorbidity status was determined with Charlson Comorbidity Index (CCI).

**Results:**

In total, 11 020 patients were included. Mean follow-up time was 7.3 years. SMRs for the six groups (no appointments, one appointment, MMCI quartiles) were 2.46 (95% confidence interval [CI] = 2.24 to 2.71), 3.55 (95% CI = 3.05 to 4.14), 1.15 (95% CI = 1.06 to 1.25), 0.97 (95% CI = 0.89 to 1.06), 0.92 (95% CI = 0.84 to 1.01) and 1.21 (95% CI = 1.11 to 1.31), respectively. With continuous MMCI, mortality formed a U-curve. The inflection point was at a MMCI value of 0.65 with corresponding SMR of 0.86. Age and CCI aHR for death between men and women was 1.45 (95% CI = 1.35 to 1.58).

**Conclusion:**

Patients with no CoC had the highest mortality. In patients having care over time, the effect of GP-CoC on mortality was minor and mortality increased with high GP-CoC.

## How this fits in

Relational continuity of care (CoC) has been shown to reduce mortality in many chronic conditions across both primary and secondary care settings; however, patients outside CoC have often been excluded from studies evaluating relational CoC. Additionally, there have been varying results on the effect of relational CoC on mortality in patients with type 2 diabetes (T2D) in primary care. This study shows high mortality in patients with T2D with no COC and, compared with that in patients with care over time, changes in mortality with different levels of relational CoC were minimal. Moreover, particularly in patients aged ≥60 years with T2D, better relational continuity of care might first be associated with reduction in mortality followed by increase in mortality with high continuity.

## Introduction

Primary health care (PHC) is a cornerstone of a sustainable and well-performing healthcare system.^1,2^ In a world of growing burden of non-communicable diseases, PHC’s potential of providing good health at low cost becomes more significant.^3^ One important driver for increasing burden is diabetes.^4^ The burden of older patients with type 2 diabetes (T2D) is expected to double by the end of 2020s.^5^

Continuity of care (CoC) is a key component of PHC and one of the core values of effective GP–patient relationships.^6,7^ Care of an individual patient and care over time conceptualise CoC. Various types of continuity — such as relational, informational, and management— have been delineated.^8^ Better physician relational CoC has been associated with reduced mortality.^9–12^ However, two previous studies on GP continuity of care (GP-CoC) and mortality in patients with T2D reported varying findings.^13,14^ An Israeli study showed reduced mortality with better GP-CoC^13^ while an Austrian study found no association between GP-CoC and mortality.^14^

Because of the longitudinal concept of CoC and indexes that are used to measure relational CoC, exclusion criteria of a minimum number of appointments have been widely used in studies examining relational CoC and mortality. In two prior systematic reviews on physician relational CoC and mortality, 15 of 26 studies measured CoC with an index. Of those 15 studies, 12 studies used an exclusion criteria of a minimum number of appointments.^9,10^ This enhances selection bias and potentially creates non-representative cohorts. In studies on PHC patients with T2D, the Israeli study excluded 22% and the Austrian study 2.5% of patients for not having enough appointments.^13,14^

Local healthcare system influences both the accessibility and CoC. This study was conducted in the public PHC of the city of Vantaa in Finland. Vantaa is located in the Helsinki metropolitan area and had a population of 228 166 at the end of 2022.^15^ In Finland, health centres responsible for public PHC are funded and maintained by municipals through taxes. During this study, there were seven health centers in Vantaa. In addition to PHC, the public sector also includes secondary health care. There is also a private sector in Finland, which is further divided into private PHC and occupational health care. In the private sector, people cover the costs themselves, through voluntary insurance, or have them covered by their employer. Employed individuals primarily use occupational health care, and those who were previously cared for in occupational health care mostly switch to public PHC after retirement. At the end of the inclusion period, the average retirement age in Finland was 61–62 years.^16^

The aim of this study was to examine the effect of no CoC, CoC as care over time, and GP-CoC on mortality in PHC patients with T2D aged ≥60 years without a requirement of a minimum number of appointments.

## Method

This cohort study examined patients aged ≥60 years with T2D within the public PHC of the city of Vantaa, Finland. The study period was between 1 January 2002 and 31 December 2018.

Owing to changes made to PHC in the city of Vantaa during the study period, where a ‘restricted-list GP model’ was launched in place of a ‘named GP model’ on 1 September 2011,^17^ the inclusion period was set between 1 January 2002 and 31 August 2011, and the follow-up period was set between 1 September 2011 and 31 December 2018. Because changes in the organisation of the local healthcare services impact on the assessment of relational CoC, it was decided to start the follow-up for all patients from the launch of the new GP care model, rather than starting follow-up for each individual from the day they were identified in the study data. Patients were followed until the end of the follow-up period or date of death, whichever occurred first.

Patients were defined as having T2D if they had at least one recording of an International Classification of Diseases (ICD-10) code E11* or a prescription of at least one antihyperglycemic drug coded according to the Anatomical Therapeutic Codes (ATC)-codes A10*. Patients were included in the study cohort if they had a diagnosis of T2D made during the inclusion period, and if they were at least 60 years old and alive at the end of inclusion period. Inclusion criterion of at least 60 years of age was used since this age group had reached normal retirement age at the time the inclusion period ended. Also, this age group mainly uses public PHC services.^18,19^ No minimum number of appointments was required for the inclusion. There were no exclusion criteria.

The following data were obtained from an electronic health record system (Finnstar): age, sex, diagnosis, number of GP appointments, GP with whom the appointment was made to, and drug prescriptions. Data on date of death were obtained from Statistics Finland.^20^

GP-CoC was measured using Modified, Modified Continuity Index (MMCI). MMCI was calculated for the follow-up period.^21^ MMCI ranges from 0–1 and higher values indicated better GP-CoC. In comparison with some other CoC indexes, such as Bice–Boxerman CoC index^22^ (COCI) and Usual Provider of Care index^23^ — and as an advantage of MMCI — the formula of MMCI does not set conditions for a minimum number of appointments. The study cohort was divided into six groups according to either the number of appointments or the MMCI quartiles. Patients with zero and one appointment, who had no CoC, formed a group each and patients with two or more appointments, who had CoC as care over time and varying degree of GP-CoC, were divided into four groups according to MMCI quartiles. Comorbidity status was determined with Charlson Comorbidity Index (CCI), based on data from the inclusion period.^24^ Mortality was assessed using adjusted hazard ratio (aHR) and standardised morality ratio (SMR).

Data are presented as means with standard deviation (SD) or as counts (*n*) with percentages (%). Group differences were investigated through a series of one-way analysis of variances (ANOVA) and χ^2^ test. Adjusted Kaplan–Meier cumulative mortality rates were estimated using inverse probability weighting (IPW); 95% confidence intervals (CIs) were obtained by bias-corrected bootstrapping (5000 replications); adjustments were made for age, sex, and CCI. Cox proportional hazard regression was used to estimate the aHRs and their 95% CIs. The ratio of observed to expected number of deaths, the SMR for all-cause deaths, was calculated using subject-year methods with 95% CI. The expected number of deaths was calculated based on sex, age, and calendar-period-specific mortality rates in the Finnish population. Non-linear trends of SMR were displayed using restricted cubic spline curves estimated by a multivariable-adjusted Poisson regression model. We used 4-knots placed at the 5th, 35th, 65th, and 95th percentiles of the cumulative MMCI distribution. Stata (version 17.0) was used for the statistical analyses.

## Results

In total, 11 020 patients were included and followed for 71 898 person-years with a mean follow-up time of 7.3 years. During follow-up, 2682 (24%) patients died. Table 1 shows the characteristics of the study cohort and crude mortality at the end of follow-up in the six groups. Groups differed in sex, mean age, SMR, and comorbidity status. The groups with zero and one appointments had lower proportion of women and were older than the MMCI groups, and the group with zero appointment had the lowest observed CCI value. Mortality in MMCI groups was close to the mortality of general population.

**Table 1.** Characteristics of the study patients aged ≥60 years who were diagnosed with type 2 diabetes (*n* = 11 020) within the primary health care of the city of Vantaa, Finland, and crude mortality at the end of follow-up in the formed six groups. Test of similarity between groups was conducted when applicable

	No appointments	One appointment	Modified, Modified Continuity Index (MMCI), quarters				
	*n =* 992	*n* = 315	Q I (MMCI<0.56) *n* = 2418	Q II (MMCI 0.56– 0.70) *n* = 2433	Q III (MMCI 0.71–0.83) *n* = 2424	Q IV (MMCI>0.83) *n* = 2438	*P* value^a^
Women, *n* (%)	446 (45)	146 (46)	1158 (48)	1243 (51)	1265 (52)	1245 (51)	<0.001
Age, mean (SD)	72 (8)	71 (9)	69 (8)	70 (7)	70 (7)	70 (7)	<0.001
Charlson Comorbidity Index, mean (SD)	1.36 (0.85)	1.65 (1.23)	1.53 (1.05)	1.64 (1.18)	1.73 (1.20)	1.73 (1.28)	<0.001
Number of appointments, per person-years (95% CI)	..	0.21 (0.19 to 0.23	1.45 (1.43 to 1.47)	2.70 (2.68 to 2.73)	3.73 (3.70 to 3.76)	3.44 (3.41 to 3.47)	<0.001
Follow-up, person-years	5567	1507	16 008	16 507	16 512	15 797	..
Crude mortality end of follow-up, percentage (95% CI)	44.0 (40.9 to 47.1)	52.1 (46.7 to 57.8)	22.2 (20.6 to 24.0)	19.7 (18.2 to 21.3)	19.4 (17.9 to 21.1)	24.4 (22.7 to 26.1)	<0.001
SMR (95% CI)	2.46 (2.24 to 2.71)	3.55 (3.05 to 4.14)	1.15 (1.06 to 1.25)	0.97 (0.89 to 1.06)	0.92 (0.84 to 1.01)	1.21 (1.11 to 1.31)	<0.001

^a^ *P* value between groups. CI = confidence interval. Q = quartile. SD = standard deviation. SMR = standardised mortality ratio

**Figure 1.**
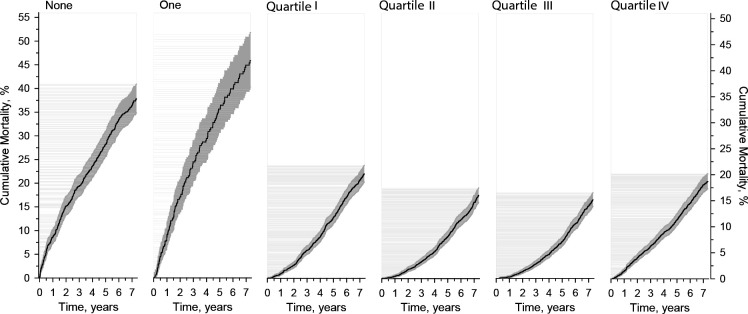
Age, sex, and Charlson Comorbidity Index adjusted cumulative mortality in the study patients aged ≥60 years who were diagnosed with type 2 diabetes within the primary health care of the city of Vantaa, Finland, divided into six groups, according to either the number of appointments (no appointments or one appointment during the follow-up period) or the quarters of the Modified, Modified Continuity Index. Grey area represents 95% confidence intervals

Figure 1 shows cumulative mortality in the six groups. Mortality in groups with both zero and one appointment were higher than mortality in MMCI groups.

**Figure 2.**
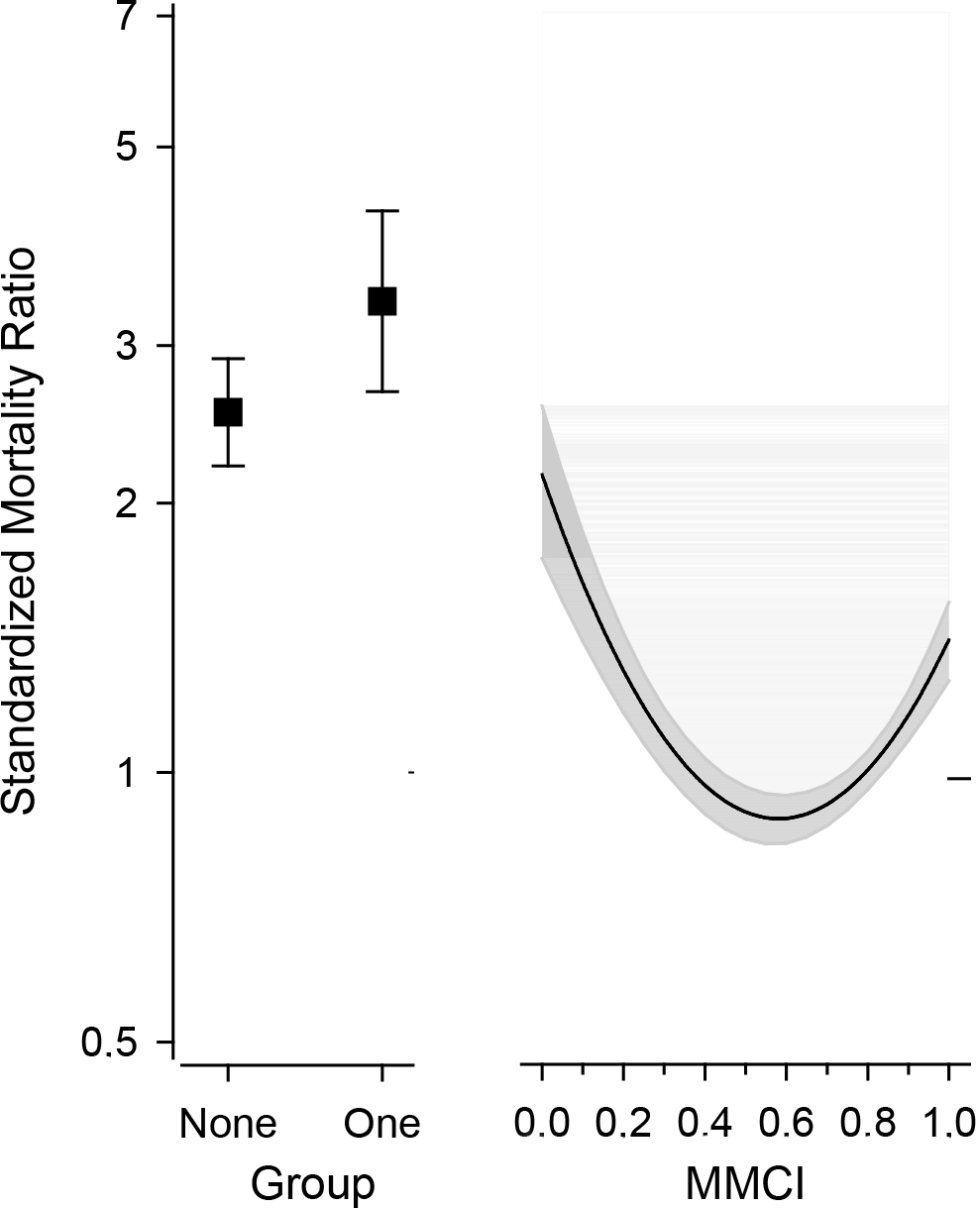
Standardised mortality ratio in the study patients aged ≥60 years who were diagnosed with type 2 diabetes within the primary health care of the city of Vantaa, Finland. Divided into no appointments or one appointment during the follow-up period or the continuous of the Modified, Modified Continuity Index (MMCI). Non-linear trends of SMR were displayed using restricted cubic spline curves estimated by a multivariable-adjusted Poisson regression model. We used 4-knots of continuous MMCI placed at the 5th, 35th, 65th, and 95th percentiles of the cumulative MMCI distribution. The restricted cubic spline models were adjusted for age, sex, and Charlson Comorbidity Index. Whiskers and grey area represent 95% confidence intervals

Figure 2 shows mortality in the groups with zero and one appointment and with continuous MMCI. With continuous MMCI and mortality, the inflection point of the U-curve was at a MMCI value of 0.65 with corresponding SMR of 0.86.

Table 2 shows aHRs for death in women, men, and the entire study cohort in the six groups compared with the group with zero appointments. All groups significantly differed in mortality from the group with zero appointments. The study cohort had excess mortality compared with the general population with a SMR of 1.23. SMR for women and men were 1.26 and 1.20, respectively, with no significant difference between the sexes (data not shown). Age and CCI aHR for mortality between men and women was 1.45 (95% CI = 1.35 to 1.58).

**Table 2.** Age, sex, and Charlson Comorbidity Index adjusted hazard ratios for death in the study patients by sex aged ≥60 years who were diagnosed with type 2 diabetes within the primary health care of the city of Vantaa, Finland, divided into six groups, according to either the number of appointments (no appointments or one appointment during the follow-up period) or the quarters of the Modified, Modified Continuity Index (MMCI). Hazard ratio of group with no appointments was used as reference group

	Women hazard ratio (95% CI)	Men hazard ratio (95% CI)	All hazard ratio (95% CI)
No appointments	1.00 (Reference)	1.00 (Reference)	1.00 (Reference)
One appointment	1.34 (1.03 to 1.77)	1.35 (1.06 to 1.72)	1.34 (1.12 to 1.60)
MMCI, Q I	0.36 (0.30 to 0.44)	0.40 (0.34 to 0.48)	0.38 (0.34 to 0.44)
MMCI, Q II	0.30 (0.25 to 0.36)	0.31 (0.26 to 0.37)	0.30 (0.26 to 0.34)
MMCI, Q III	0.27 (0.22 to 0.33)	0.28 (0.23 to 0.34)	0.27 (0.24 to 0.31)
MMCI, Q IV	0.35 (0.29 to 0.42)	0.40 (0.33 to 0.47)	0.37 (0.33 to 0.42)
*P* for linearity	<0.001	<0.001	<0.001

CI = confidence interval. Q = quarter

## Discussion

### Summary

In patients with T2D aged ≥60 years in the public PHC of the city of Vantaa, the highest mortality was observed in patients with zero or one appointment (patients who had no CoC). In patients with T2D who had care over time (patients with at least two appointments during the follow-up period), mortality was altogether markedly lower compared with patients with T2D who had no CoC, and the effect of GP-CoC on mortality was minor. In patients with care over time, mortality first reduced with better GP-CoC but increased with high GP-CoC. The study cohort had excess mortality compared with the general population with no difference between the sexes. Mortality was higher in men compared with women. This study does not provide insight into the mechanisms by which care over time and GP-CoC affect mortality, but plausible mechanisms include enhanced patient–physician relationship, improved understanding of patients’ health needs, better management and preventive care, and increased adherence to treatment.

### Strengths and limitations

This study has strengths regarding study setting and parameters used. Inclusion of all patients with T2D without a requirement of a minimum number of appointments enhances the representativeness of the intended patient group. Additionally, the representability of the study cohort is bolstered by the inclusion of patients aged ≥60 years, who mainly use public PHC services, and study’s location in a single city with public PHC accessible to all citizens. Utilisation of SMRs expands evaluation of mortality to a population level. Considering the comprehensive nature of PHC appointments, inclusion of all appointments, irrespective of the reason for visits, provides a good view of GP care received. The used mortality data are valid coming from Statistics Finland.

Limitations of this study should be acknowledged. The study focused on GP appointments, leaving effects of relational CoC with other healthcare professionals unknown. One-fifth of patients with T2D in Finland are aged <60 years^25^ and the results presented here may not apply to these patients. Regarding the representativeness of the study cohort, it is possible that some patients with T2D, who were otherwise eligible for the inclusion, did not use public PHC services during the inclusion period and were thus not captured by this study. Owing to the chosen study method, a cohort study, confounding factors, such as exposures to care and CoC before the beginning of the follow-up period, remain unclear. The study data did not include information on certain lifestyle factors, such as smoking or physical activity levels, which could have significant implications. Data on social and economic conditions were not available, and thus this study is unable to examine the potential effect of these conditions on patients’ care-seeking characteristics and mortality. Certain comorbidities that could impact mortality, such as hypertension, are not included in CCI^24^ and were thus left outside analysis. Owing to diagnosis recording habits and limitations related to the electronic health record system from which study data were gathered, only one recording of an ICD-10 code E11* was required for a patient to be defined as having T2D, which may affect the cohort’s representativeness. Information on the duration of T2D and whether T2D was complicated would have provided valuable insights, but these data were not available. Lack of detailed information on diseases from national registers leaves some observed differences, such as the low CCI value among patients with zero appointments compared with the other groups, open for further studies. Study data did not include residency data and thus emigration was not assessed.

### Comparison with existing literature

In this study, CoC, as having care over time, resulted in a significant reduction in mortality (*P* value <0.001) and the effect of GP-CoC on mortality was minor. In patients having care over time, mortality first decreased with better GP-CoC but an increase in mortality was found with high GP-CoC values. These are novel findings in PHC patients with T2D. Aforementioned Austrian and Israeli studies excluded patients with few appointments and potentially ignored a high mortality group with no CoC.^13,14^ The Israeli study reported reduction in mortality in a high GP-CoC group while an Austrian study found no association between GP-CoC and mortality and showed no rise in mortality with high GP-CoC. There are differences between this study and the two studies (such as patient age, length of follow-up time, cohort size, and methods of measuring continuity and mortality) but notably, in the Austrian study, 62% of the study cohort had a COCI value of 1. High percentage of patients seeing only one GP, attributable to the local organisation of healthcare services, mostly explains the findings in the Austrian study. Differences in GP-CoC might not influence health indicators,^26^ which could explain the minor effect of GP-COC on mortality found in this study. Also, the rise in mortality with high GP-CoC that was found in the present study could result from factors not assessed in this study, such as severity of T2D, complications of T2D needing frequent GP care, or GPs' behavioural habits regarding, for example, referrals to secondary care. In PHC patients with T2D, CoC as having care over time, might produce more significant mortality reduction compared with better GP-CoC. Also, in older PHC patients with T2D, mortality could increase with high GP-CoC.

Better physician CoC in PHC and secondary care has been associated with reduced mortality in various age groups and in different chronic conditions.^9,10,12,27^ Of the studies conducted in PHC, only one study, examining patients aged ≥70 years in the US, did not use a minimum number of appointments as an exclusion criterion.^28^ In the US study, patients with no CoC had the highest mortality and the effect of GP-CoC on mortality was minor.^28^ Although the US study did not assess GP-CoC continuously, the reported aHRs compared with the group with no CoC increased with high GP-CoC. Although existing evidence has shown a reduction in mortality with a better relational CoC, based on results from this study and the US study, in older PHC patients, having CoC as care over time seems to provide most of the mortality reduction compared with changes in GP-CoC. The findings of this study also suggest that strict exclusion criterion based on the number of appointments may overlook patients with poorer outcomes who do not have any CoC.

Mortality in older patients with T2D was higher compared with the general population. The observed excess mortality is consistent with findings from previous studies in patients with T2D conducted in Sweden and the UK.^29,30^ These two studies also reported a decrease in excess mortality with ageing, which was not explored in our study. Although excess mortality decreased with older age, it was still present in the older age groups in the Swedish study. The UK study presented excess mortality categorised by age and duration of diabetes, revealing varying findings of decreased, increased, and unchanged excess mortality in older patients. Generally, it appears that patients aged ≥60 years with T2D have excess mortality compared with the general population. However, some subgroups, particularly patients aged ≥75 years with longer durations of T2D, may not exhibit excess mortality.

Mortality in men was higher compared with women, and no difference in excess mortality between the sexes was found in this study. The aforementioned Austrian study reported male sex as a significant predictor for mortality^14^ and a UK study found higher mortality rate in men compared with women.^30^ A Swedish nationwide study and a Danish population-based study reported no difference in excess mortality between the sexes.^31,32^ Differing from this study and the Swedish and Danish studies, studies from Scotland and the UK reported more excess mortality in women compared with men.^33,34^ It seems that men with T2D have higher overall mortality and at least comparable excess mortality compared with women.

### Implications for research and practice

The present study showed the highest mortality in patients with no CoC. Further studies encompassing other disease cohorts are warranted to examine whether such a high risk mortality group exists beyond T2D. For future research, since excess mortality in T2D decreases with ageing,^29,30^ it could be beneficial to study younger patients with T2D and examine whether a similar and potentially more prominent high mortality group with no CoC exists. This study implicates that in clinical practice, mortality could be reduced by identifying and engaging patients with no CoC, thereby integrating them into the protective framework of GP care. Considering the findings of this study, when implementing enhanced relational CoC within healthcare services, organisations should acknowledge that vulnerable and morbid individuals may exist beyond the bounds of CoC.

In conclusion, in patients aged ≥60 years with T2D in a public PHC setting, the highest mortality was observed in patients without any CoC. In patients with care over time, mortality was lower compared with patients with no CoC, the effect of GP-CoC on mortality was minor and the initially observed decrease in mortality with better GP-CoC turned into an increase with high GP-CoC. The study cohort had excess mortality compared with the general population and men’s mortality was higher compared with women.

## Data

Sharing the research data of this study is prohibited for ethical and legal grounds. The license applicant may only use data from the Finnish Institute for Health and Welfare, Statistics Finland, and the Social Insurance Institution for the purposes specified in the license agreement. As a result, the data cannot be shared with third parties. If such a request is made, all permissions must be reapplied for.
